# Protective effect of PCV vaccine against experimental pneumococcal challenge in adults is primarily mediated by controlling colonisation density

**DOI:** 10.1016/j.vaccine.2019.05.080

**Published:** 2019-07-09

**Authors:** E.L. German, C. Solórzano, S. Sunny, F. Dunne, J.F. Gritzfeld, E. Mitsi, E. Nikolaou, A.D. Hyder-Wright, A.M. Collins, S.B. Gordon, D.M. Ferreira

**Affiliations:** a Liverpool School of Tropical Medicine, Liverpool, United Kingdom; b Royal Liverpool University Hospital, Liverpool, United Kingdom

**Keywords:** Pneumococcus, PCV, Colonisation, Density, QPCR

## Abstract

Widespread use of Pneumococcal Conjugate Vaccines (PCV) has reduced vaccine-type nasopharyngeal colonisation and invasive pneumococcal disease. In a double-blind, randomised controlled trial using the Experimental Human Pneumococcal Challenge (EHPC) model, PCV-13 (Prevenar-13) conferred 78% protection against colonisation acquisition and reduced bacterial intensity (AUC) as measured by classical culture. We used a multiplex qPCR assay targeting *lytA* and pneumococcal serotype 6A/B *cpsA* genes to re-assess the colonisation status of the same volunteers. Increase in detection of low-density colonisation resulted in reduced PCV efficacy against colonisation acquisition (29%), compared to classical culture (83%). For experimentally colonised volunteers, PCV had a pronounced effect on decreasing colonisation density. These results obtained in adults suggest that the success of PCV vaccination could primarily be mediated by the control of colonisation density. Studies assessing the impact of pneumococcal vaccines should allow for density measurements in their design.

## 1 Introduction

Pneumonia is a leading cause of death in children under 5 years worldwide, causing up to 1.4 million deaths annually [1]. Of these deaths, approximately 38% are caused by *Streptococcus pneumoniae* (pneumococcus) [2]. Current licensed pneumococcal conjugate vaccines (PCVs) are highly effective in protecting against invasive pneumococcal diseases caused by vaccine-type serotypes in children [3]. Immunization with PCVs also has beneficial indirect effects, conferring herd immunity to unvaccinated adults [4]. Disease and transmission prevention requires interruption of colonisation [3]. Numerous studies have reported a reduction in vaccine-type (VT) pneumococcal colonisation acquisition after PCV vaccination, however, the impact of PCVs on pneumococcal colonisation density is less clear [5], [6], [7], [8]. Studies have shown that vaccination with PCV reduces colonisation densities with VT serotypes when comparing to control vaccines [5]. However, other studies have reported that vaccination with PCV do not affect pneumococcal density in children colonised by VT serotypes [7].

We have previously reported the results of a double-blind, randomised controlled trial investigating the effect of the 13-valent PCV (PCV-13, Prevnar-13, Pfizer) on pneumococcal colonisation using the Experimental Human Pneumococcal Challenge (EHPC) model [9]. PCV showed 78% protection against acquisition of pneumococcus serotype 6B (BHN418) (5/48 became colonised in PCV arm vs 23/48 in the control arm). Moreover, although the number of volunteers who became experimentally colonised following PCV vaccination was limited, we observed a significant reduction in nasal pneumococcal colonisation density (3 log difference in PCV arm compared to control arm at day 2) [9].

Interest in using molecular methods for colonisation detection is increasing because of their high sensitivity and, therefore, their ability to detect pneumococcus at low colonisation densities. Combining the results of classical culture and molecular methods has been suggested in order to improve accuracy of reported colonisation rates [10]. The *lytA* (autolysin) gene qPCR strategy developed by the Centers for Disease Control (CDC) is currently the WHO-recommended culture-independent method to detect pneumococci [11]. However, given the capacity of pneumococcus to exchange genes with other streptococci [12] a multiplex approach is valuable. Therefore, in this study, we employed a multiplex qPCR assay targeting *lytA* and pneumococcal serotype 6A/B *cpsA* genes to re-assess volunteer samples from our PCV study for experimental colonisation of 6B pneumococcus. We compared the results obtained by this molecular method with the results previously obtained by classical culture.

## 2 Material and methods

### 2.1 PCV/EHPC clinical trial

A trial investigating the efficacy of the 13-valent PCV vaccine against experimental human pneumococcal challenge was conducted in 2012. Study design and outcomes have been previously reported [9]. Briefly, 96 healthy volunteers aged 18–50 were vaccinated with either PCV-13 (PCV arm, n = 48) or Hepatitis A vaccine (control arm, n = 48). 4–5 weeks post-vaccination, volunteers were inoculated with 80,000 Colony-Forming Units (CFU) per nostril of live 6B pneumococcus (BHN418, sequence type 138) [9]. Nasal wash samples were taken and processed to obtain a nasal wash supernatant and a nasal wash bacterial pellet as described previously [13], before and after pneumococcal inoculation (at days 2, 4, 14 and 21). Samples were stored at −80 °C. This trial was approved by The National Health Service Research and Ethics Committee (REC) (12/NW/0873 Liverpool) and was co-sponsored by the Liverpool School of Tropical Medicine and the Royal Liverpool and Broadgreen University Hospitals Trust. Informed consent was obtained from all volunteers.

### 2.2 DNA extraction

300 μl of D2, D7 and D14 nasal wash bacterial pellets was centrifuged for 7 min at 20,238*g*. Following centrifugation, 300 μl of lysis buffer with protease, 100 μl of Zirconium beads (Stratech, Ely, UK) and 300 μl of Phenol (Sigma-Aldrich, St Louis, MO, USA) was added to the pellet and the sample was disrupted twice for 3 min in a tissue homogenizer (Bertin Technologies, Montigny le Bretonneux, France) followed by 3 min on ice. The sample was centrifuged for 10 min at 9391*g*, and the upper aqueous phase was transferred to a tube pre-filled with 600 μl binding buffer and 10 μl magnetic beads. The samples were incubated at room temperature for 30–120 min then washed twice with 200 μl of wash buffers 1 and 2. Magnetic beads were dried at 55 °C for 10 min, eluted in 63 μl of elution buffer and stored at −20 °C. Lysis buffer, protease, binding buffer, magnetic beads, wash buffers 1 and 2, and elution buffer are part of the Agowa mag Mini DNA isolation kit (LGC Genomics, Berlin, Germany).

### 2.3 Multiplex qPCR

We developed a novel multiplex qPCR based on methods previously published, using partial amplification of *lytA* [11] and 6A/B *cpsA* [14] genes. The sequences of the primers and probes used are: *lytA* forward primer: 5′-ACG-CAA-TCT-AGC-AGA-TGA-AGC-A-3′; *lytA* reverse primer 5′-TCG-TGC-GTT-TTA-ATT-CCA-GCT-3′; *lytA* probe: 5′-(FAM)-TGC-CGA-AAA-CGC-TTG-ATA-CAG-GGA-G-(BHQ-1)-3′; *cpsA* forward primer: 5′-AAG-TTT-GCA-CTA-GAG-TAT-GGG-AAG-GT-3′; *cpsA* reverse primer: 5′-ACA-TTA-TGT-CCA-TGT-CTT-CGA-TAC-AAG-3′; *cpsA* probe: 5′-(HEX)- TGT-TCT-GCC-CTG-AGC-AAC-TGG-(BHQ-1)-3′. The reaction mixture of 25 µl contained 0.6 µM of each *lytA* primer, 0.3 µM of *lytA* probe, 0.4 µM of each *cpsA* primer, 0.2 µM of *cpsA* probe, 12.5 µM of Taqman Gene Expression Master Mix (Applied Biosystems, USA) and 2.5 µl of extracted DNA. The qPCR reaction was run on a Mx3005P machine (Agilent Technologies, Santa Clara, CA, USA) on the following programme: 10 min at 95 °C followed by 40 cycles of 15 s at 95 °C and 1 min at 60 °C. DNA from BHN418 serotype 6B, extracted using the QIAamp DNA mini kit (Qiagen, Hilden, Germany) and serially diluted 1:10 from 4.14 × 10^6^ copies in 2.5 µl, was used as a standard curve. A sample was considered positive if at least one duplicate had a CT value less than 40. The 40 cycle cut-off correlates well with classical culture data where it is known whether or not viable serotype 6B pneumococcus has been isolated.

### 2.4 Statistical analysis

Risk Ratio, 95% confidence interval (CI) and p-value were calculated. Colonisation densities were determined from number of gene copies per well and analysed in GraphPad Prism v5 (GraphPad Inc.). Densities were log-transformed and means with standard deviations were calculated. Unpaired t-tests were used to compare densities in PCV and control arms.

## 3 Results

### 3.1 Colonisation acquisition rates by molecular methods

Samples from 90 of the 98 volunteers enrolled in the EHPC PCV study were available and included in these analyses. The re-assessment of the PCV/EHPC trial samples using the developed multiplex qPCR showed that all volunteers reported as colonisation-positive (acquired the inoculated bacterium) by classical culture were also found to be colonised with pneumococcus by molecular methods. However, the number of colonisation-positive volunteers increased in both PCV and control arms when using molecular methods*.* Positivity at any day for both *lytA* and *cpsA* was found in 22/45 (49%) volunteers in the PCV arm and 31/45 (69%) volunteers in the control arm (Table 1). The risk ratio by classical culture was 0.17 (95% CI, 0.07–0.46; P = 0.0005) and by molecular methods was 0.71 (95% CI, 0.50–1.01; P = 0.06). PCV conferred 83% protection against experimental pneumococcal colonisation by classical culture, and 29% protection by molecular methods.

**Table 1:** Comparison of numbers of colonised volunteers by detection method, study day and study arm.

	Classical Culture No. Colonised/Total No. (%) [9]		*lytA*/*cpsA* multiplex qPCR No. Colonised/Total No. (%)	
	PCV	Control	PCV	Control
D2	2/38 (5)	18/39 (46)	11/38 (29)	21/39 (54)
D7	4/43 (9)	18/42 (43)	16/43 (37)	23/41 (56)
D14	1/9 (11)	18/23 (79)	2/9 (22)	17/23 (74)
Any Day	4/45 (9)	23/45 (51)	22/45 (49)	31/45 (69)
Risk Ratio (p-value)	0.17 (0.0005)		0.71 (0.06)	

Definition of abbreviations: PCV = pneumococcal conjugate vaccine; NW = nasal wash.

When assessing the breakdown of colonisation-positive volunteers per study day, the percentage of experimentally colonised volunteers by *lytA*/*cpsA* multiplex qPCR was similar between D2 and D7 regardless of study arm (Table 1).

### 3.2 Colonisation densities by molecular methods

**Fig. 1:**
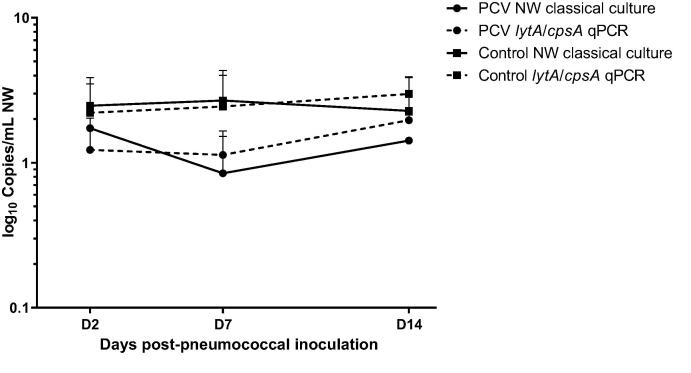
Colonisation densities by vaccination group, detection method and study time-point. Values shown are the log_10_ mean copies/mL ± SD. Classical culture data has been previously reported [9].

Colonisation densities were significantly lower in volunteers vaccinated with PCV compared to the control arm by both classical culture (P = 0.03) and molecular methods (P < 0.0001) (Fig. 1, Table 2).

**Table 2:** Comparison of colonisation density of colonised volunteers by detection method, study day and study arm.

	Classical Culture Log_10_ CFU/ml Mean ± SD [9]		*lytA*/*cpsA* multiplex qPCR Log_10_ DNA copies/ml Mean ± SD	
	PCV	Control	PCV	Control
D2	1.73 ± 0.02	2.47 ± 1.39	1.22 ± 0.80	2.21 ± 1.29
D7	0.85 ± 0.67	2.70 ± 1.64	1.13 ± 0.52	2.44 ± 1.56
D14	1.42	2.28 ± 1.58	1.86 ± 0.16	2.98 ± 0.93

Definition of abbreviations: PCV = pneumococcal conjugate vaccine; CFU = colony forming units; SD = standard deviation; NW = nasal wash.

There was a significant correlation between densities calculated by classical culture and by *lytA*/*cpsA* qPCR for volunteers in both PCV (P < 0.0001; r^2^ = 0.54) and control arms (P < 0.0001; r^2^ = 0.85). 91% of samples positive by *lytA*/*cpsA* qPCR but not by classical culture had densities < 30 DNA copies/ml. There is no significant difference between mean densities calculated by classical or molecular methods, either in the PCV or in the control arm (Table 2). By either detection method, densities in the PCV arm were significantly lower (P = 0.0010 by classical culture and P < 0.0001 for molecular methods) than in the control arm.

## 4 Discussion

In this study we re-assessed the experimental colonisation status of all volunteers from our PCV/EHPC trial using a multiplex qPCR. The number of volunteers that became colonised after experimental pneumococcal inoculation by molecular methods was higher than the number previously reported by classical culture [9]. This increase in number is more pronounced in the PCV arm (23 vs 4 volunteers) than in the control arm (31 vs 23 volunteers), which translates into increased risk ratios, and therefore a decrease in the calculated protective efficacy of PCV against pneumococcal colonisation from 83% to 29%.

Molecular methods such as qPCR can detect DNA from dead bacteria. However, it is unlikely that this phenomenon had a major contribution to our findings. If this method were detecting remains of the pneumococcal inoculum administered on Day 0, we would expect to see higher colonisation rates at D2 than D7. However, the colonisation rates on both days are comparable.

The *cpsA* gene sequence used in our multiplex qPCR is common to both serotypes 6A and 6B. However, epidemiological studies have reported that the incidence of pneumococcal serotype 6A is very low in the UK in the post-PCV era; this was confirmed by screening 795 volunteers for the EHPC studies in Liverpool which found only one volunteer carrying a serotype 6A [15]. Pneumococcal capsular genes have recently been discovered in other streptococci [16], [17], raising the possibility that the additional gene copies detected by qPCR derive from non-pneumococcal streptococci. Nevertheless, in the context of the EHPC model, screening samples are taken before inoculation, carriage is assessed very soon post-inoculation and volunteers have limited contact with children. The most likely explanation for the detection of *lytA* and *cpsA* genes in our multiplex qPCR is carriage of the experimental 6B pneumococcus used.

Both classical culture and molecular detection methods demonstrate a lower colonisation density in volunteers vaccinated with PCV. The additional colonised volunteers detected in both arms by molecular methods are mostly colonised at a low density. It has been suggested that colonisation density plays an underestimated but pivotal role in the development of pneumococcal disease and in transmission dynamics [18], [19]. Our results further support the hypothesis that colonisation density is a determining factor for the clinical outcome and spread of *S. pneumoniae*. It is plausible that a novel vaccine that controlled pneumococcal colonisation density independently of any effect on pneumococcal acquisition would be effective in reducing pneumococcal disease. Measures of colonisation density, by both classical culture and molecular methods, should therefore be accounted for in the design of vaccine efficacy trials, as was demonstrated recently in a Phase 2 trial of a novel pneumococcal vaccine [20].

Findings from previous epidemiological studies have proved inconclusive regarding the impact of PCVs on pneumococcal colonisation density [5], [6], [7], [8]. The advantage of the EHPC model is the ability to study colonisation dynamics in a controlled manner, clarifying causality and eliminating the effect of many confounding factors. Its main disadvantage is the ethical imperative to use adult volunteers rather than children. It is possible that the effect of PCV on colonisation density differs between adults and children, and our findings will need to be validated in younger cohorts.

## 5 Conclusions

Using molecular methods, we have shown that PCV conferred 29% protection against experimental colonisation acquisition. This may indicate that the main protective mechanism of this vaccine is mediated by reduction of colonisation density, leading to a decreased risk of disease to vaccinated individuals as well as transmission resulting in the observed herd effects in vaccinated populations.

## Funding

This work was supported by the Medical Research Council/FAPESP (grant number MR/M011569/1), Medical Research Council (grant number MR/M011569/1), the Bill & Melinda Gates Foundation, (grant numbers OPP1035281 and OPP1117728); and the National Institute for Health Research (NIHR) Local Clinical Research Network. The funders were not involved in the design, data processing or publication of this work.

## Author contributions

ADH-W, AMC, SBG and DMF designed and co-ordinated the PCV clinical trial; JFG, EM and DMF processed clinical samples; ELG, CS, SS, FD, JFG, EM and EN performed DNA extractions and qPCRs; ELG, CS, EM and EN developed the multiplex qPCR; ELG, CS and DMF analysed the data and drafted the manuscript. All authors have read and approved the manuscript.

This work was partially presented as a poster at the 10th International Symposium on Pneumococci and Pneumococcal Diseases (ISPPD-10), 26th-30th June 2016, abstract number 330.

## Declaration of Competing Interest

None.
